# Editorial: Polyphenols as potent modulators of inflammation-associated non-communicable diseases

**DOI:** 10.3389/fnut.2026.1782023

**Published:** 2026-01-29

**Authors:** Abdelhafid Nani, Aziz Hichami

**Affiliations:** 1 Faculty of Natural and Life Sciences, University of Ahmed Draia, Adrar, Algeria; 2 Physiologie de Nutrition & Toxicology (NUTox), UMR UB/INSERM 1231 Center for Cellular & Translational Molecular Medicine (CTM), Université Bourgogne Europe Dijon, Dijon, France

**Keywords:** polyphenols, health, inflammation, non-communicable diseases, mechanisms

Polyphenols constitute a diverse class of phytochemicals, with more than 8,000 characterized structures, predominantly derived from plant sources such as cereals, fruits, and vegetables. These compounds are notable for their phenolic structures and numerous hydroxyl groups, which characterize their antioxidant capacities (1). The complexities of their absorption in the small intestine, subsequent metabolism within the liver and intestines, and the transformative functions of gut microbiota in the colon underscore their intricate biological roles (2). Although advancements have been made in understanding physiological states such as pregnancy, lactation, and aging, as well as pathophysiological conditions, especially non-communicable diseases (NCDs) such as inflammatory bowel diseases (IBDs), pulmonary disorders, and cardiovascular ailments, the effects of polyphenols on these inflammation-related conditions remain only partially understood.

In this Research Topic on *Polyphenols as Potent Modulators of Inflammation-Associated Non-Communicable Diseases*, we received original research and review articles that thoroughly examined the multi-target mechanisms by which phenolic-rich healthy diets and traditional herbs influence various physiological conditions.

In their original research article, Yu et al. investigated how *Aurantii Fructus* extract (AFE), a derivative of unripe citrus fruit, serves as a therapeutic agent for ulcerative colitis by targeting specific molecular pathways and the gut microbiome. Through a murine study, the authors demonstrated that AFE treatment significantly reduces colon damage and physical symptoms by inhibiting the NF-κB inflammatory pathway and activating the Nrf2/HO-1 antioxidant pathway. Beyond chemical signaling, the extract strengthened the intestinal mucosal barrier by protecting goblet cells and restoring essential tight junction proteins. Finally, the study highlighted AFE's ability to restore microbial homeostasis, specifically by suppressing pathogenic bacteria, such as *Proteobacteria*, while fostering a diverse beneficial gut environment. Together, these findings suggested that AFE is a multi-targeted natural intervention capable of alleviating oxidative stress and dysbiosis, which are central to chronic intestinal diseases.

A pilot study by Fiecke et al. investigated how a mother's genetic secretor status influences the way her diet changes the bioactive composition of her breast milk. Researchers provided a Mediterranean-style meal plan to breastfeeding women and tracked changes in carotenoids, polyphenols, and oligosaccharides, finding that a healthy diet significantly increased specific health-promoting metabolites primarily in women with the maternal α1,2-fucosyltransferase 2 (FUT2) secretor phenotype. While dietary carotenoid levels remained stable despite higher intake, the concentrations of beneficial polyphenol metabolites and certain complex sugars were differentially modulated by underlying maternal genetics. The study suggested that non-dietary factors, such as genetics and the gut microbiome, are essential for understanding the highly variable nutritional profile of human milk. These findings underscored the personalized nature of nutrition and have significant implications for optimizing maternal diets to support infant health.

The narrative review by Shao et al. examined salvianolic acid B (SalB), the primary bioactive component of the traditional Chinese herb *Salvia miltiorrhiza*, and evaluated its potential as a modern cardioprotective agent. SalB's primary biological activities include potent antioxidant, anti-inflammatory, anti-fibrotic, anti-thrombotic, and anti-apoptotic properties. SalB was identified as a multi-target natural compound that exerts its wide-ranging biological effects by modulating an intricate network of signaling pathways. Its pharmacological actions are not isolated but interact synergistically, enhancing its therapeutic potential for complex diseases. The regulatory network of SalB demonstrated significant cross-talk. For example, it regulated the AKT/mTOR pathway for antioxidant, anti-inflammatory, and anti-cancer effects while also attenuating the CD36/PI3K/AKT pathway for anti-fibrotic actions. It also synergistically inhibited oxidative stress and inflammation through regulating the Nrf2/NLRP3 and NF-κB/NLRP3 pathways.

Preclinical research has demonstrated SalB's efficacy across a broad spectrum of cardiovascular conditions, including atherosclerosis, myocardial infarction (MI), myocardial ischemia-reperfusion injury (MI/RI), cardiac hypertrophy, and various forms of cardiomyopathy. However, the clinical translation of SalB is hindered by significant challenges, primarily its structural instability and extremely low oral bioavailability.

A meta-analysis by Wu et al., encompassing 15 randomized controlled trials (RCTs) with 894 participants, explored the clinical efficacy and safety of dietary polyphenols as adjunctive treatments for chronic obstructive pulmonary disease (COPD). The study demonstrated that phenolic compounds had varying effects. Indeed, curcumin and salidroside (from Salvia) significantly reduced systemic inflammation and improved respiratory function. Globally, dietary supplementation with eight polyphenols led to a significant overall reduction in pro-inflammatory cytokines (IL-6, TNF-α) and a significant increase in IL-10 anti-inflammatory cytokine. Salidroside and curcumin demonstrated the most significant and consistent benefits. Salidroside was effective in improving coagulation markers, lung function, TNF-α, and symptom scores. Curcumin significantly reduced systolic blood pressure. The study provided evidence that these bioactive supplements can modulate metabolic-inflammatory networks, lowering blood pressure and improving lung parameters, such as Forced Expiratory Volume in 1 second (FEV1) and the FEV1/Forced Vital Capacity (FVC) ratio. Hence, the authors advocated for personalized nutritional interventions to transform polyphenol preparations into precise, cost-effective therapies for lung disease management.

Overall, the themes in this Research Topic highlighted the therapeutic potential of plant-derived compounds for the management of inflammation-associated NCDs. Dietary polyphenols modulated multiple interconnected pathways through:

- Inhibition of the NF-κB, a central regulator of inflammation.
- Activation of the Nrf2/HO-1 pathway, which is crucial for cellular defense against oxidative stress.
- Modulation of gut microbiota by strengthening the intestinal mucosal barrier and restoring microbial homeostasis.
- Activation of the AMPK pathway, which is increasingly recognized for its diverse effects on inflammatory signaling beyond its established role in metabolic regulation of food intake.
